# Gene Systems Network Inferred from Expression Profiles in Hepatocellular Carcinogenesis by Graphical Gaussian Model

**DOI:** 10.1155/2007/47214

**Published:** 2007-07-26

**Authors:** Sachiyo Aburatani, Fuyan Sun, Shigeru Saito, Masao Honda, Shu-ichi Kaneko, Katsuhisa Horimoto

**Affiliations:** 1 Biological Network Team, Computational Biology Research Center (CBRC), National Institute of Advanced Industrial Science and Technology (AIST), 2-42 Aomi, Koto-ku, Tokyo 135-0064, Japan; 2 Chemo & Bio Informatics Department, INFOCOM CORPORATION, Mitsui Sumitomo Insurance Surugadai Annex Building, 3-11, Kanda-Surugadai, Chiyoda-ku, Tokyo 101-0062, Japan; 3 Department of Gastroenterology, Graduate School of Medical Science, Kanazawa University, 13-1 Takara-machi, Kanazawa, Ishikawa 920-8641, Japan

## Abstract

Hepatocellular carcinoma (HCC) in a liver with advanced-stage chronic hepatitis C (CHC) is induced by hepatitis C virus, which chronically infects about 170 million people worldwide. To elucidate the associations between gene groups in hepatocellular carcinogenesis, we analyzed the profiles of the genes characteristically expressed in the CHC and HCC cell stages by a statistical method for inferring the network between gene systems based on the graphical Gaussian model. A systematic evaluation of the inferred network in terms of the biological knowledge revealed that the inferred network was strongly involved in the known gene-gene interactions with high significance , and that the clusters characterized by different cancer-related responses were associated with those of the gene groups related to metabolic pathways and morphological events. Although some relationships in the network remain to be interpreted, the analyses revealed a snapshot of the orchestrated expression of cancer-related groups and some pathways related with metabolisms and morphological events in hepatocellular carcinogenesis, and thus provide possible clues on the disease mechanism and insights that address the gap between molecular and clinical assessments.

## 

[1,2,3,4,5,6,7,8,9,10,11,12,13,14,15,16,17,18,19,20,21,22,23,24,25,26,27,28,29,30,31,32,33,34,35,36,37,38]
